# Implicit Motor Imagery for Chronic Pelvic Pain: A Cross-Sectional Case–Control Study

**DOI:** 10.3390/jcm12144738

**Published:** 2023-07-18

**Authors:** Esther Díaz-Mohedo, Gloria González-Roldán, Inmaculada Muñoz-Gámez, Virginia Padilla-Romero, Eduardo Castro-Martín, Irene Cabrera-Martos, Clara Sánchez-García

**Affiliations:** 1 Department of Physiotherapy, Faculty of Health Sciences, University of Malaga, Avda. Arquitecto Francisco Peñalosa, s/n, 29071 Málaga, Spain; 2 Faculty of Health Sciences, University of Málaga, 29071 Málaga, Spain; 3 Department of Physiotherapy, Faculty of Health Sciences, University of Granada, 18016 Granada, Spain

**Keywords:** chronic pain, graded motor imagery, pelvic pain, body schema, laterality discrimination, brain changes

## Abstract

Implicit motor imagery (IMI), with an image laterality discrimination (LD) task, has been proposed as a useful therapeutic tool to restore body schema in patients with chronic pelvic pain (CPP). The aim of this study was to analyse the existence of differences between patients with CPP and healthy individuals in order to justify the use of IMI. An observational, cross-sectional study with non-probabilistic sampling was designed as a one-to-one matched case–control study. Through a web link designed for this purpose, a total of 40 abdominoperineal images were shown to 130 participants during the laterality task. Outcome measures were pain intensity (visual analogue scale, VAS), accuracy, response time (RT), and CPPQ-Mohedo score (Chronic Pelvic Pain Questionnaire—Mohedo). This was an observational, cross-sectional study with a total of 64 CPP patients and 66 healthy individuals. The comparative analysis between groups revealed significant differences in accuracy, CPPQ-Mohedo and VAS (*p* < 0.001), but not in RT; in patients with CPP, accuracy was correlated with a lower CPPQ-Mohedo score and RT and, the greater the pain intensity, the higher the CPPQ-Mohedo score and RT, and the lower the accuracy. In the LD task, the patients with CPP made more mistakes than the healthy individuals. IMI could be a useful and complementary tool in the therapeutic approach for patients with CPP.

## 1. Introduction

Chronic pelvic pain (CPP) is defined as “the chronic or persistent pain that is perceived in the structures related to the pelvis in men and women”. It often coexists with symptoms that suggest a lower urinary tract, sexual, intestinal, pelvic floor, or gynaecological dysfunction. When associated with negative cognitive, behavioural, sexual, and emotional consequences, it is known as CPP syndrome [1].

CPP has been described as a global public mental health priority and it is among the most prevalent healthcare problems (14.8% in older women over 25 years of age) [1,2] and economic issues [3,4,5]. Moreover, it causes a decrease in the quality of life, followed by an increase in morbidity and mortality [1,3,6].

According to the International Classification of Diseases [7], although its origin could be a documented nociceptive pain, it becomes chronic when it has been continuous or recurrent for at least three months [3] or, in the case of cyclic pain, over six months. Several studies have shown that, during such time, a cortical reorganisation takes place, causing structural, morphological, and functional changes in the central nervous system (CNS) [4,5,6,8,9,10,11,12,13,14,15,16,17,18], specifically in the pain modulation areas, which can maintain the perception of pain in the absence of acute injuries, with alteration of the sensory processing and greater emotional/cognitive processing of pain [12,19].

Such cortical reorganisation is characterised by an increase in the excitatory neural activity, along with a decrease in the inhibitory neural activity, in addition to alterations in the amount of grey matter in the anterior insula, amygdala [20], primary somatosensory cortex (S1), and hippocampus. These changes generate and maintain situations such as: increase in pain perception [13,17,21], changes in the integration of the viscerosomatic and motor process [22,23], and the appearance of maladaptive cognitive and emotional components (catastrophising, kinesiophobia, depression and alteration of attention and decision making in external tasks) [11,24]. All these characteristics are pathognomonic and representative in patients with CPP.

In view of such a complex clinical reality, to date, the first line of treatment for CPP is pharmacological therapy, with surgical intervention being the last choice, with little success. Currently, there is increasing support in the literature that shows that chronic pain is associated with the interruption of the cortically supported body schema [25,26,27,28,29]. A practical way of exploring the integrity of the body schema and its influence on the symptoms of these patients would be through graded motor imagery (GMI).

GMI is defined as a non-pharmacological and non-invasive treatment constituted by three phases conducted gradually and in an orderly manner and aimed at treating certain painful conditions. It was created and developed by David S. Butler and G. Lorimer Moseley during the first decade of the 21st century, when a better understanding of the interactions between the brain, the peripheral tissues, and the environment of the patient required new therapeutic approaches that addressed the brain areas involved in the painful movement [30,31].

Initially, GMI uses implicit motor imagery (IMI), with a laterality discrimination (LD) task. This first stage assesses the capacity of the individual to evaluate whether the body image observed belongs to the right or left side. During this task, both the accuracy (number of correct answers) and the RT (response time: time spent to provide the correct answer) are analysed, since these parameters are potentially altered in many painful conditions, both acute and chronic.

In healthy people, response times are proportional to the time taken to physically move their physical body part to match the image [32] and are slower for images depicting more awkward positions, reflecting biomechanical constraints, and consistent with the use of IMI [33]. Longer-than-normal response times are thought to reflect delayed neural processing or decreased cortical weighting of a given body part or spatial zone [34]. Poor accuracy is thought to indicate disrupted cortical proprioceptive representation of the body part [34,35].

Jackson et al. demonstrated that internal mental images produce major sensorimotor cortical activity [36]. Along these lines, it has been shown that the degree of mental effort during IMI tasks influences the cortical activity, such that greater effort leads to greater hemodynamic changes in the brain [37].

A recent systematic review revealed that poor motor imagery performance on the LD was a common feature of chronic peripheral musculoskeletal pain conditions [33], and that it remains unknown as to whether chronic pelvic pain is similarly affected.

These findings suggest that determining IMI performance in people with chronic pelvic pain is warranted. If IMI is found altered in people with chronic pelvic pain, this may indicate disruption of the working body schema, improving the understanding of the condition, and it may provide the opportunity for novel treatment methods such as graded motor imagery.

The current therapeutic approach for CPP is based on the biopsychosocial model, and it proposes multimode interventions, which are insufficiently implemented in the public healthcare system.

Inspired by these findings and motivated by the innovation of mobile health in chronic diseases, we proposed to improve the available therapies for CPP, creating and developing a mobile application.

We are currently immersed in the development of an app for these patients, in which, in addition to offering resources of therapeutic education regarding pain and advice on therapeutic exercise (both procedures recommended in the Clinical Practice Guidelines on the approach for chronic pain), we explored the usefulness of including IMI techniques as an added functionality. Thus, it was necessary to carry out this study, whose results help us to estimate its possible utility as a complementary therapeutic tool.

Since this app is currently being developed, the present study only includes the LD task, through a web link that was designed for this purpose.

We hypothesised that CPP would be associated with delayed responses and/or reduced accuracy of the abdominoperineal LD task. Our secondary hypothesis was that pain intensity in patients with CPP would be negatively related to parameters on the abdominoperineal LD task (accuracy and RT), age, and CPPQ-Mohedo score.

The aim of the study was, using a pelvic floor LD, in people with and without CPP:

1. to explore the existence of significant differences between groups measured in percentage of accuracy and RT;
2. to determine the existence of correlation between pain intensity in patients diagnosed with CPP and: (a) age, (b) the CPPQ-Mohedo questionnaire score, (c) the number of correct answers in the LD task of such images, and (d) RT in such discrimination.

## 2. Materials and Methods

### 2.1. Study Design

An observational, cross-sectional study with non-probabilistic sampling was conducted to assess the ability to identify and discriminate pelvic floor images among chronic pain patients with non-chronic pain patients as the control group (CG). The study was designed as a one-to-one matched case–control study. Each participant was paired with at least one participant of the same sex and age (±5 years). The design followed the international recommendations for Strengthening the Reporting of Observational Studies in Epidemiology [38]. The data were collected between May 2022 and July 2022. A written informed consent was obtained from each of the participants before their inclusion. All the participants received an explanation of the study procedures, which were planned according to the ethical standards of the Declaration of Helsinki. Ethical approval was received from the Bioethics Committee of the Medical University of Málaga (17-2022-H).

### 2.2. Participants

A non-probabilistic sampling by convenience was performed, obtaining a sample of 26 patients from ADOPEC (Chronic Pelvic Pain Association in Spain), 38 patients referred by health professionals, and 66 volunteers for the control group recruited through the patients and/or professionals themselves, as well as through social networks (CG).

The inclusion criteria for the CPP patients were as follows: (a) having a clinical diagnosis of CPP [1], (b) Spanish-speaking men and women of legal age, (c) and having a score of over 6 points in the CPPQ-Mohedo questionnaire [39]. The inclusion criteria for the CG were as follows: (a) having no diagnosis of CPP, (b) Spanish-speaking men and women of legal age, (c) and having a score of over 6 points in the CPPQ-Mohedo questionnaire. The following exclusion criteria were common to both groups: (a) being diagnosed with dyslexia, (b) having previously undergone motor imagery training, and (c) failing to perform the test correctly.

### 2.3. Outcome Measures

#### 2.3.1. Pain Intensity

A visual analogue scale (VAS) [40] was used to measure pain intensity before each test. The VAS comprises a horizontal line from 0 (representing ‘no pain’) to 10 (representing ‘pain as bad as you can imagine’). The participants placed a mark on the line at the point where they perceived that their pain intensity was represented at the time of the test, which was quantified by the application. This scale has proved its reliability and validity for the measurement of pain intensity.

#### 2.3.2. Accuracy

The number of correct responses was expressed as a percentage over the total number of pictures displayed [33].

#### 2.3.3. Response Time

This is the length of time taken to answer whether a picture is right or left. The mean RT spent to complete the test was expressed in minutes and seconds [33].

#### 2.3.4. CPPQ-Mohedo

A questionnaire to help discriminate between patients with and without CPP symptoms (sensitivity and specificity: 0.968; reliability: Cronbach’s alpha, 0.75) was used, with a cutoff point of 6 [39].

All variables were collected through an electronic link.

### 2.4. Procedure

Control task: left/right judgements of photographs of the abdominoperineal area.

The participants undertook the left/right abdominoperineal area judgement task using the established protocol. Five hundred and fifty photographs of the right and left side, in different positions and divided by sexes, were employed in random order, with the participants accessing these photographs through a web link to part of the software of an app in the process of development and implementation (App-Mohedo^®^, SAVE CREATIVE (20/04/2020). COD: 2004213727578). The term sex is used as a classification of male or female based on biological distinction of genitalia throughout the manuscript. A total of 40 images were shown to each participant [41]. The participants responded by pressing one button on their mobile phone if the photograph showed a left abdominoperineal area and a different button if it showed a right abdominoperineal area. Emphasis was placed on the speed and accuracy of the responses. That is, the participants were instructed to make accurate responses as quickly as possible. The task consisted of a single test.

#### Protocol

Via videocall with a member of the research team, the participants were invited to make themselves comfortable before taking the test. Then, through the chat, they were given the electronic link that granted them access to the questionnaire, in which they provided their basic information and completed the GMI test. Prior to the test, they watched a short video that explained the task. Once they watched it, the researcher asked them whether they were ready and invited them to place their thumbs on the appropriate response keys for the left and right sides [41]. The participants then performed the left/right abdominoperineal area judgement task. Throughout the test, the researcher accompanied the participants telematically to ensure that they were not helped, distracted, or performing the task in the wrong way.

### 2.5. Statistical Analysis

All statistical analyses were performed using Jamovi (2021) and R Core Team (2022) [42,43].

Background variables are presented as means with a standard deviation and ranges or frequencies and percentages. Student’s *t* tests were used to analyse differences between sexes, and Pearson’s linear correlation to explore the linear association between the percentage of correct answers and RT in the images. The Chi-squared test was used to explore the association between the qualitative variables. The level of significance was defined at *p* < 0.05.

## 3. Results

The total study sample consisted of 147 participants, of whom 17 patients were excluded for being diagnosed with dyslexia. Finally, 130 participants met the inclusion criteria and were evenly divided into two groups (64 CPP patients and 66 CG).

There were no statistically significant differences in the sociodemographic data between the two groups, as is shown in Table 1 (*p* > 0.05).

The comparative analysis between groups revealed significant differences in accuracy, CPPQ-Mohedo, and VAS variables (*p* < 0.001), but not in RT; these results are shown in Table 2.

Table 3 shows the results of the Pearson correlation analysis. It shows that: (1) age was significantly correlated with RT and VAS; (2) VAS was significantly correlated with RT, accuracy, and CPPQ-Mohedo; and (3) accuracy was significantly correlated with RT.

Therefore, it can be deduced that accuracy is correlated with a lower CPPQ-Mohedo score and RT, and the greater the pain intensity measured with VAS and age, the higher the CPPQ-Mohedo score and RT, and the lower the accuracy.

## 4. Discussion

To the best of the authors’ knowledge, this is the first study to associate IMI with CPP.

The results obtained in this study revealed statistically significant differences between the two groups (case and control) in CPPQ-Mohedo questionnaire score, VAS, and accuracy; that is, the case group obtained greater scores in pain intensity and lower accuracy in the LD task compared to the control group. There were no differences with respect to the time spent to complete the image discrimination task.

Furthermore, there was a significant correlation between several variables. On the one hand, the greater the age, the longer the time spent to complete the task and the greater the pain intensity; similarly, the larger the number of correct answers, the shorter the time spent to complete the task (better performance). Lastly, a greater pain intensity was significantly correlated with a greater score in the discriminant questionnaire, lower accuracy, and longer RT in the LD task.

The results were in line with those obtained in similar studies, although the latter were conducted on other lethal chronic conditions, such as complex regional pain syndrome [44], phantom limb pain [45,46], and chronic back pain [41,47,48], showing a correlation between chronic pain and the interruption of the cortically supported body schema. In such pathologies, these studies reported a distorted perception of the body image and size and orientation of the specific painful area.

Taking the mentioned studies as reference, one of the aspects that were considered for the present study was the question of whether GMI would be applicable in this case, given the absence of the concept of spatial laterality in the pelvic floor (patients with CPP perceive pain in the entire pelviperineal area), as well as its representation in the cortex compared to the other locations.

Currently, the cortical somatosensory representation of the genitals in women and men is still poorly understood, and contradicting results have been reported.

Since the study of Penfield and Rasmussen in 1950 [49], which was based on electrical stimulation during open brain surgery, there has been a lack of data on the cortical somatosensory representation of the genitals. Then, through the use of electromyography techniques, nuclear magnetic resonance, and transcranial stimulation, these data have been further detailed with studies that support the interpretation that there are two different representations of the genital sensations in S1: one in the medial surface and the other in the lateral surface. Moreover, it is suggested that the secondary somatosensory cortex and the posterior insula support a representation of the affective aspects of the genital sensation [50].

Regarding pelvic floor musculature control, the key areas of the brain involved are the primary motor cortex, the supplementary motor area, the cingulate gyrus, the putamen, the thalamus, the prefrontal cortex, the supramarginal gyrus, the insula, and the cerebellum [51,52].

The mentioned studies have verified the representation and activation of such cortical areas through the visualisation of erotic images or application of tactile stimulation in the genital area or through the request for voluntary muscle contraction and control of urination in healthy individuals, respectively [51,53].

In CPP, a motor cortical alteration of the pelvic floor has been described, showing that this is an important study area, which could also predict how the patient would respond to the treatment. For instance, in recent neuroimaging studies at rest, with respect to healthy controls, the patients with chronic pelvic pain exhibited an altered frequency content of the obtained functional magnetic resonance imaging (fMRI) of a specific motor region of the pelvis and an altered connectivity between said motor region and other regions of the brain [54]. Furthermore, in recent brain morphology studies, compared to healthy controls, the patients with chronic pelvic pain showed significant differences in the volume of grey matter and the diffusion anisotropy in motor cortical areas in line with the control of such musculature [55].

In the present study, although this interruption of the body schema, specifically of the pelvic floor, has not been evidenced to date, owing to the lack of imagery techniques that refute our results, the cortical alteration appeared slightly in the smaller number of correct answers (or more mistakes) obtained during the task performed in our case group, with respect to the CG. It is important to highlight that bad sexual experiences or sexual abuse are strongly associated with CPP [56], and thus it is recommended to carefully record this aspect throughout the clinical history of patients with CPP. It is known that the cortical representation of the genitals has its maximal expression during puberty, and it is later modulated by “good or bad” sexual experiences [53]. Children who have suffered sexual abuse show neuroplastic adaptation, resulting in an altered cortical representation of sensory and processing areas that are directly involved in the abusive experience, and these adverse sensory experiences might lead to impaired neurodevelopmental and thus structural changes in adulthood [57]. However, on the other hand, it is known that a positive genital self-image is positively related to sexual desire and positive body perception [58].

In addition to the sensitive part that comprises the genital area, it is known that there is poor proprioception of the pelvic muscles, which can be attributed to the lack of visual stimuli and the scarce joint movement during contraction. This proprioceptive deficit is combined with women’s lack of knowledge about their pelvic and genital area [59].

It is clear that the LD task of GMI with the images presented to the sample of this study required the activation of “possibly affected” brain areas, although this cannot confirm the influence of a proprioceptive deficit, poor self-image, or bad sexual experiences. It can only assert that these participants found it more difficult to plan, adjust, automatise, and voluntarily execute the postures and/or movements shown in the images. It would be interesting for future studies to gather these variables and estimate their relationship with the results obtained in the present study.

### 4.1. Response Time (RT)

Regarding the lack of significance with respect to RT, the results were in line with those of other authors [41,47], who did not detect significant differences in RT in the left/right discrimination task between the cases and the controls for chronic lumbar pain, which is similar to CPP in terms of the centred spatial location of pain (non-unilateral). Interestingly, this does not occur in unilateral chronic limb pain (specifically, the shoulders) [60,61]; these authors found that the number of correct answers was not significant, although significance was obtained for RT. This is attributed to the fact that, at the limb level, the information processing towards the healthy side is probably biased, which would produce an erroneous initial automatic selection; however, this selection would be corrected when the information is transmitted to the hemisphere of the healthy side, thus increasing RT, understanding it as the additional time required to correct the erroneous initial judgement but not the incorrect answers during the GMI test [47]. In any case, this is a hypothesis that needs further research in this regard.

These results of RT in our study could be partly explained by the lack of knowledge, poor identification, and self-perception of the pelvic and genital area in the general population (both patients and healthy individuals), and thus the poor motor control of the pelvic floor musculature. For example, in a previous study [62], 98% of healthy women thought that they contracted their pelvic floor musculature correctly and voluntarily, although only 33% of them achieved this; a different study found a low level of knowledge of the pelvic floor musculature in the general population [63]. In any case, and in order to reject or accept this hypothesis, it would be convenient to use the scales of self-image knowledge and genital self-perception in patients with CPP, which would allow determining the existence of a relationship between the alterations in the perception of the pelvic and/or genital area and CPP.

### 4.2. Accuracy

In this study, this variable showed a statistically significant difference between groups, which was in line with the findings of other authors, who, in addition to reporting such differences between groups (chronic cervical pain vs. acute cervical pain/no pain) [64], demonstrated that there were no significant differences between groups in terms of correct answers when the LD task was conducted with the same groups, but using images of another body part (hand) [65]. Such results suggested the existence of a body schema disturbance (S1) related to the affected zone, which influences the variable of correct answers in the execution of the LD task.

### 4.3. Age

Regarding age, the results were in line with those reported in chronic shoulder pain by Breckenridge JD et al. [33] and Ravat S et al. [66], showing that the greater the age, the smaller the number of correct answers (accuracy) and the longer the RT to complete the LD test.

Natural ageing involves a physiological defect in the somatosensory system, from the receptors, with a decrease in all sensory sources, to the processing at the CNS.

When brain activity is analysed through functional magnetic resonance, it can be observed that, with age, there is a decrease in functional connectivity between different brain regions, including the posterior cingulate cortex, the praecuneus, the medial prefrontal cortex, and the lateral parietal cortex, which systematically show a decrease in activation compared to the baseline during the execution of the task [67].

Landelle et al. [68] analysed the age-related degenerative proprioceptive changes during the induction of unilateral illusory sensations, comparing young adults and older adults (over 65 years of age); they found that, the proprioceptive stimulus of the right hand produced a significant decrease in the contralateral S1 (left) activation and a lower deactivation of the ipsilateral homologous S1 (right), with respect to the baseline level in the group of older adults, inducing an imbalance between the two cortexes, which caused an alteration in the coding of the kinematic, proprioceptive, and perceptive parameters of the body and movement. According to Lebel C et al., this is due to a decrease in the size of the corpus callosum with age, which hinders communication between the two hemispheres [69], specifically the laterality tasks. All this could justify the results of this study.

### 4.4. CPPQ-Mohedo

CPPQ-Mohedo [39] has proved to be a very useful discriminant tool in the evaluation of patients with CPP [70], since it allowed us to establish a positive diagnosis of the patients that constituted the group of cases, as well as to determine whether any of the volunteers in the control group could be a “non-diagnosed case”. The cutoff point used was the one suggested by its authors (case ≥ 6 points in the questionnaire). The score in this questionnaire had a positive correlation with that obtained in VAS, as was expected.

### 4.5. Pain

Regarding pain, a positive correlation between pain intensity and RT was found, as well as a negative correlation between pain intensity and accuracy in the test (number of correct answers); thus, the greater the pain intensity of the lumbar-pelvic-perineal area, the smaller the number of correct answers and the longer the RT; this was in line with the results of other authors in their studies on low back pain [33,48,71], who also showed that patients with pain found it more difficult and required more time to complete these mental tasks [71].

Pain intensity is a very complex variable in constant controversy, as it can vary in patients with CPP and influence their performance in the task depending on many more variables [60,64], such as the origin of pain, coexistence with another chronic disorder [60], recurrence [41], depression, anxiety, self-efficacy [72,73], level of catastrophising [74,75,76], affective distress, and disability [77], etc.

However, several authors [60,78] stated that such factors are independent from the results in the laterality tests.

These results related to pain intensity raise questions on whether pain intensity could be an essential factor to be taken into account when determining the suitability of IMI as a complementary therapeutic tool in the approach for CPP.

To date, no guide or protocol has been published to specify the correct procedure to evaluate the results of the IMI task (recommended number of images, sessions to be conducted, previous training, timing, difficulty progression, etc.). Only one study [60] was found in the literature whose authors used 80 images in the LD task and performed a previous training session before gathering their data, and another study in which the patients were considered to be disrupted with slow response times (>2.5 s, and reduced accuracy (<80%)) [79]. Further research is necessary in this regard to develop an effective therapeutic protocol.

The use of IMI with the LD task has proved to be a reliable, non-invasive method with no adverse effects, which makes it a suitable evaluation and complementary technique in the approach for any chronic pain [47], including CPP.

App-Mohedo is a tool under development and implementation that has been developed for mobile devices, and which, in addition to accessing therapeutic resources (e.g., therapeutic education on pain and advice on therapeutic exercise), will allow evaluating and training-improving the capacity of the individual to assess whether the observed image of the pelviperineal area belongs to the left or right side of the body. During this task, both the accuracy (number of correct answers) and RT (response time) are analysed, which are parameters that showed potential alteration in painful conditions such as CPP. The aim is to inform, with further studies, whether such training influences the modulation and decrease in pain intensity and the functional improvement of these patients, which is the end goal of this project.

### 4.6. Bias

During sample collection, comorbidity with other pathologies for which the patients were receiving another treatment at the moment of the study was not taken into account. In the case of women, they were matched up based on whether they had given birth, although disregarding the number of births.

On the other hand, the researcher was aware at all times of the group that each participant belonged to.

### 4.7. Limitations

Because of its characteristics, the use of IMI depends on each particular individual’s capacity for imagination, as well as on the level of the self-image of the genital area. The present study did not use any tool that evaluated this aspect.

Since the study was conducted online, it was not possible to quantify the pelvic floor musculature motor control in the participants and, therefore, it was not possible to correlate the results with a possible motor control deficit.

The small number of patients included in the final sample, after applying our criteria, was a limitation. The results were conclusive, although; with a larger sample size, more statistically significant results could be achieved.

## 5. Conclusions

In the IMI laterality discrimination task performed in the sample:

1. There are significant differences in the number of correct answers between CPP patients and the CG: the patients made more mistakes than the healthy individuals.
2. The greater the pain intensity of the patients diagnosed with CPP, the greater the score in the CPPQ-Mohedo questionnaire, the smaller the number of correct answers, and the longer the time required to complete the LD task with pelvic floor images. The greater the age of the patients, the longer the RT and the greater the score in the CPPQ-Mohedo.

IMI can be a useful and complementary tool to help, with pelvic floor images, to activate and reorganise the cerebral cortex and its inhibitory mechanisms in patients with CPP. It will be necessary to reproduce the study in a larger sample to obtain more powerful statistical results.

## Figures and Tables

**Table 1:** Age and sex of the case and control groups.

	Control (N = 66)	Case (N = 64)	Total (N = 130)	*p* Value
AGE				0.332 ^1^
Mean (SD)	45.1 (12.0)	47.0 (9.6)	46.0 (10.9)	
Range	22.0–76.4	27.2–70.7	22.0–76.4	
SEX				0.620 ^2^
Female	45.0 (68.2%)	41.0 (64.1%)	86.0 (66.2%)	
Male	21.0 (31.8%)	23.0 (35.9%)	44.0 (33.8%)	

^1^ = Linear Model ANOVA, ^2^ = Pearson’s Chi-squared test.

**Table 2:** Comparative analysis between groups.

	Control (N = 66)	Case (N = 64)	Difference	*p* Value
RT				0.222 ^1^
N-Miss	3.0	1.0		
Mean (SD)	3.0 (1.3)	3.3 (2.2)	-0.3 (0.3)	
Range	1.6–8.3	0.4–15.6		
Accuracy				0.025 ^1^
N-Miss	3.0	1.0		
Mean (SD)	88.9 (10.8)	83.6 (15.0)	5.2 (2.3)	
Range	37.5–100.0	42.5–100.0		
(CPPQ)-Mohedo				<0.001 ^1^
Mean (SD)	1.3 (1.6)	9.0 (1.7)	−7.6 (0.2)	
Range	0.0–6.0	7.0–16.0		
VAS				<0.001 ^1^
Mean (SD)	1.3 (2.8)	6.9 (2.1)	−5.6 (0.4)	
Range	0.0–10.0	0.0–10.0		

^1^ = Linear Model ANOVA, RT: Response time; VAS: Visual analogue scale; CPPQ-Mohedo: chronic pelvic pain questionnaire (CPPQ)-Mohedo.

**Table 3:** Results of the Pearson correlation analysis.

Correlation Matrix						
		Age	RT	Accuracy	CPPQ-Mohedo	VAS
AGE	Pearson’s r	—				
	*p*-value	—				
RT	Pearson’s r	0.333 ***	—			
	*p*-value	<0.001	—			
ACCURACY	Pearson’s r	−0.169	−0.211 *	—		
	*p*-value	0.058	0.018	—		
CPPQ-Mohedo	Pearson’s r	0.100	0.091	−0.136	—	
	*p*-value	0.258	0.312	0.129	—	
VAS	Pearson’s r	0.176 *	0.181 *	−0.237 **	0.800 ***	—
	*p*-value	0.045	0.042	0.007	<0.001	—

* *p* < 0.05, ** *p* < 0.01, *** *p* < 0.001.

## Data Availability

Not applicable.
